# Tetra­bromidobis(dicyclo­hexyl­phosphane-κ*P*)digallium(*Ga*—*Ga*)

**DOI:** 10.1107/S1600536812035982

**Published:** 2012-09-05

**Authors:** Dennis H. Mayo, Yang Peng, Peter Zavalij, Kit H. Bowen, Bryan W. Eichhorn

**Affiliations:** a Department of Chemistry and Biochemistry, Chemistry Building 094, University of Maryland, College Park, MD 20742, USA; b Departments of Chemistry and Materials Science, Johns Hopkins University, Baltimore, MD 21218, USA

## Abstract

The title compound, a Ga^II^ dimer, [Ga_2_Br_4_(C_12_H_23_P)_2_], was synthesized by reaction of GaBr(THF)_*n*_ (THF is tetra­hydro­furan) with dicyclo­hexyl­phosphine in toluene. At 150 K the crystallographically centrosymmetric molecule exhibits disorder in which one of the two independent cyclo­hexyl groups is modelled over two sites in a 62 (1):38 (1) ratio. In *d*_6_-benzene solution, the compound exhibits virtual *C*_2*h*_ symmetry as determined by ^1^H NMR. The coordination environment of the Ga^II^ atom is distorted tetrahedral.

## Related literature

For references related to the synthesis of the ‘GaBr’ precursor and to cluster formation, see: Schnoeckel (2010 ▶); Steiner *et al.* (2004 ▶). For other Ga—Ga containing compounds, see: Baker *et al.* (2003 ▶) (the analogous digallium tetra­iodide compound); Uhl *et al.* (1989 ▶) [the first-reported Ga(II) dimer compound].
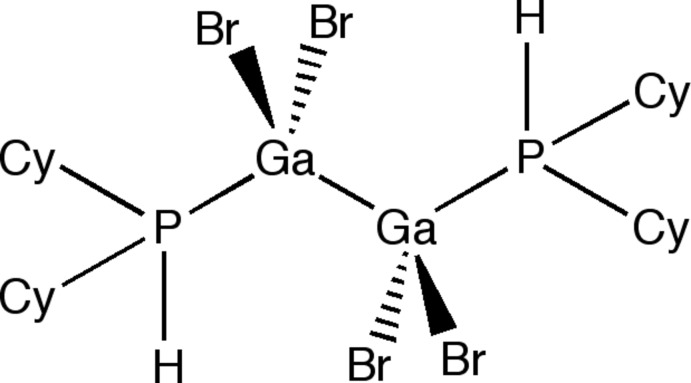


## Experimental

### 

#### Crystal data

[Ga_2_Br_4_(C_12_H_23_P)_2_]
*M* *_r_* = 855.63
Monoclinic, 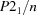

*a* = 9.6095 (11) Å
*b* = 13.7083 (16) Å
*c* = 13.3305 (16) Å
β = 109.177 (2)°
*V* = 1658.6 (3) Å^3^
*Z* = 2
Mo *K*α radiation
μ = 6.55 mm^−1^
*T* = 150 K
0.36 × 0.27 × 0.19 mm

#### Data collection

Bruker SMART APEX2 CCD area-detector diffractometer
Absorption correction: multi-scan *SADABS* (Sheldrick, 1996 ▶) *T*_min_ = 0.185, *T*_max_ = 0.288
24854 measured reflections
4842 independent reflections
4253 reflections with *I* > 2σ(*I*)
*R*_int_ = 0.021

#### Refinement

*R*[*F* ^2^ > 2σ(*F* ^2^)] = 0.034
*wR*(*F* ^2^) = 0.068
*S* = 1.00
4842 reflections
168 parameters
60 restraints
H atoms treated by a mixture of independent and constrained refinement
Δρ_max_ = 1.88 e Å^−3^
Δρ_min_ = −0.94 e Å^−3^

### 

Data collection: *APEX2* (Bruker, 2010 ▶); cell refinement: *SAINT* (Bruker, 2010 ▶); data reduction: *SAINT*; program(s) used to solve structure: *SHELXS97* (Sheldrick, 2008 ▶); program(s) used to refine structure: *SHELXL97* (Sheldrick, 2008 ▶); molecular graphics: *XSHELL* (Bruker, 2010 ▶); software used to prepare material for publication: *APEX2*.

## Supplementary Material

Crystal structure: contains datablock(s) I, global. DOI: 10.1107/S1600536812035982/nk2174sup1.cif

Structure factors: contains datablock(s) I. DOI: 10.1107/S1600536812035982/nk2174Isup2.hkl

Additional supplementary materials:  crystallographic information; 3D view; checkCIF report

## Figures and Tables

**Table:** 

Ga1—Br2	2.3612 (5)
Ga1—Br1	2.3807 (5)
Ga1—P1	2.4164 (7)
Ga1—Ga1^i^	2.4353 (6)

**Table:** 

Br2—Ga1—Br1	107.306 (18)
Br2—Ga1—P1	101.11 (2)
Br1—Ga1—P1	98.25 (2)
Br2—Ga1—Ga1^i^	114.05 (2)
Br1—Ga1—Ga1^i^	115.13 (2)
P1—Ga1—Ga1^i^	118.93 (2)

Symmetry code: (i) 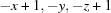.

**Fig. 1.**
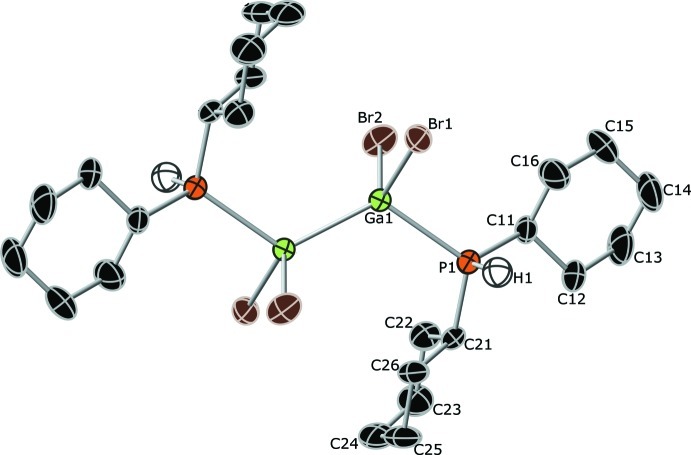
X-ray crystal structure of Ga2Br4(PHCy2)2 viewed along C2 axis. Bromine = brown, carbon = black, gallium = green, hydrogen = white, phosphorous = orange. Thermal ellipsoids shown at 50% probability; all non-phosphine hydrogen atoms removed for clarity. Non-labeled atoms are related to labeled atoms by inversion symmetry.

## supplementary crystallographic information

### Comment

GaBr(THF)_n_ was generated in a modified metal halide co-condensation reactor (Schnoeckel, 2010) at 900 K and co-condensed with a mixture of toluene:THF (3:1) at 77 K. Upon warming in the presence of dicyclohexylphosphine, the dimeric Ga_2_Br_4_(PHCy_2_)_2_ forms *via* a disproportionation reaction (Equation 1). This reaction is similar to the disproportionation of `GaI' in the presence of dicyclohexylphosphine (Baker *et al.*, 2003).

4 GaBr(THF) + 4 Cy_2_PH → Ga_2_Br_4_(PHCy_2_)_2_ + 2 Ga (Eq. 1)

Ga_2_Br_4_(PHCy_2_)_2_ exhibits 1 symmetry in the solid state, with a Ga—Ga distance of 2.435 (1) Å, but virtual C_2h_ symmetry in solution. The Ga—Br1 and Ga—Br2 distances are 2.3612 (5) and 2.3807 (5) Å, respectively; the Ga—P bond is 2.415 (3) Å. The Br—Ga—Br angle measures 107.30 (2)°.

The overall structure of Ga_2_Br_4_(PHCy_2_)_2_ is in close agreement with that of the Ga_2_I_4_(PHCy_2_)_2_ analogue reported by Baker *et al.* (2003). In Ga_2_I_4_(PHCy_2_)_2_ the Ga—Ga bond is 2.437 (1) Å; the Ga—P bonds average 2.424 (2) Å. The I—Ga—I angle in the iodo analogue is 110.07 (3)°.

The Ga—Ga distance in Ga_2_Br_4_(PHCy_2_)_2_ (2.435 (1) Å) is shorter than the 2.54 (1) Å Ga—Ga distance in the trigonal planar Ga(II) compound Ga_2_(CH(TMS)_2_)_4_ (Uhl *et al.* 1989). The Ga—Br distances in Ga_2_Br_4_(PHCy_2_)_2_ (2.370 (10) Å) are slightly shorter than the Ga—Br distances (2.4246 (22) and 2.4296 (27) Å) in the anionic [Ga_51_(P*^t^*Bu_2_)_14_Br_6_]^3-^ cluster (Steiner *et al.* 2004).

### Experimental

Ga_2_Br_4_(PHCy_2_)_2_: Dicyclohexylphosphine (2.5 mmol, 5 g of a 10% *w*/*w* solution in hexanes) was dissolved in toluene (5 ml). The solution was cooled to -78 °C and a cold (-78 °C) solution of GaBr(THF)_n_ (6.05 ml of a 380 m*M* solution in toluene:THF 3:1) was added. The resultant orange solution was stirred at -78 °C for 2 h, after which it was heated to 80 °C for 19 h. The resulting dark-brown solution was cooled to room temperature, the solvent removed *in vacuo* and the black residue dissolved in toluene (50 ml). The dark-brown solution was separated from the grey powdery residue *via* cannula filtration, concentrated, and cooled to -20 °C. After 7 d, colorless crystals of Ga_2_Br_4_(PHCy_2_)_2_ formed (40 mg, 0.047 mmol, 4% yield). ^1^H NMR (500 MHz, C_6_D_6_) δ (p.p.m.): 1.03–2.05 (44 H, Cy—H), 4.10 (dt, 2 H, 1 J(P—H) = 352 Hz, 3 J(H—H) = 5 Hz, P—H). ^13^C NMR (125 MHz) δ (p.p.m.): 25.5, 27.2, 30.3, 30.8, 31.1, 31.8. ^31^P NMR (201.6 MHz) δ (p.p.m.): -36.7 (d, J = 352 Hz).

### Refinement

One of two symmetrically independent cyclohexyl groups (C11–C16) appeared to be split in two parts tilted from each other by about 8°. The disorder of this group was refined as following: the geometry of both parts was restrained to be similar; the atomic displacement parameters (adp) were set to be the same for the same atoms in both parts, while the adp for the one cyclohexyl group was restrained to rigid-body motions and the adp were restrained to reasonable anisotropy. Total number of restraints used was 60. The occupancy of both parts was refined to be in a 0.62 (1) to 0.38 (1) ratio. H atoms were treated by a mixture of independent and constrained refinement.

### Crystal data

**Table:**

[Ga_2_Br_4_(C_12_H_23_P)_2_]	*F*(000) = 844
*M**_r_* = 855.63	*D*_x_ = 1.713 Mg m^−^^3^
Monoclinic, *P*2_1_/*n*	Mo *K*α radiation, λ = 0.71073 Å
Hall symbol: -P 2yn	Cell parameters from 12733 reflections
*a* = 9.6095 (11) Å	θ = 2.7–30.5°
*b* = 13.7083 (16) Å	µ = 6.55 mm^−^^1^
*c* = 13.3305 (16) Å	*T* = 150 K
β = 109.177 (2)°	Prism, colourless
*V* = 1658.6 (3) Å^3^	0.36 × 0.27 × 0.19 mm
*Z* = 2	

### Data collection

**Table:**

Bruker SMART APEX2 CCD area-detector diffractometer	4842 independent reflections
Radiation source: fine-focus sealed tube	4253 reflections with *I* > 2σ(*I*)
Graphite monochromator	*R*_int_ = 0.021
Detector resolution: 8.333 pixels mm^-1^	θ_max_ = 30.0°, θ_min_ = 2.2°
φ and ω scans	*h* = −13→13
Absorption correction: multi-scan (*SADABS*; Sheldrick, 1996)	*k* = −19→19
*T*_min_ = 0.185, *T*_max_ = 0.288	*l* = −18→18
24854 measured reflections	

### Refinement

**Table:**

Refinement on *F*^2^	Primary atom site location: structure-invariant direct methods
Least-squares matrix: full	Secondary atom site location: difference Fourier map
*R*[*F*^2^ > 2σ(*F*^2^)] = 0.034	Hydrogen site location: inferred from neighbouring sites
*wR*(*F*^2^) = 0.068	H atoms treated by a mixture of independent and constrained refinement
*S* = 1.00	*w* = 1/[σ^2^(*F*_o_^2^) + (0.01*P*)^2^ + 4.865*P*], *P* = (max(*F*_o_^2^,0) + 2*F*_c_^2^)/3
4842 reflections	(Δ/σ)_max_ = 0.001
168 parameters	Δρ_max_ = 1.88 e Å^−^^3^
60 restraints	Δρ_min_ = −0.94 e Å^−^^3^

### Special details

**Table:**

Geometry. All e.s.d.'s (except the e.s.d. in the dihedral angle between two l.s. planes) are estimated using the full covariance matrix. The cell e.s.d.'s are taken into account individually in the estimation of e.s.d.'s in distances, angles and torsion angles; correlations between e.s.d.'s in cell parameters are only used when they are defined by crystal symmetry. An approximate (isotropic) treatment of cell e.s.d.'s is used for estimating e.s.d.'s involving l.s. planes.
Refinement. Refinement of *F*^2^ against ALL reflections. The weighted *R*-factor *wR* and goodness of fit *S* are based on *F*^2^, conventional *R*-factors *R* are based on *F*, with *F* set to zero for negative *F*^2^. The threshold expression of *F*^2^ > σ(*F*^2^) is used only for calculating *R*-factors(gt) *etc*. and is not relevant to the choice of reflections for refinement. *R*-factors based on *F*^2^ are statistically about twice as large as those based on *F*, and *R*-factors based on ALL data will be even larger.

### Fractional atomic coordinates and isotropic or equivalent isotropic displacement parameters (Å^2^)

**Table:**

	*x*	*y*	*z*	*U*_iso_*/*U*_eq_	Occ. (<1)
Ga1	0.43950 (3)	0.06462 (2)	0.53291 (2)	0.02724 (7)	
Br1	0.58862 (3)	0.20359 (2)	0.60235 (3)	0.04156 (8)	
Br2	0.33747 (4)	0.01282 (3)	0.66273 (3)	0.04888 (9)	
P1	0.23183 (7)	0.14796 (5)	0.40942 (5)	0.02762 (13)	
H1	0.274 (4)	0.225 (2)	0.368 (3)	0.043 (9)*	
C11	0.0946 (13)	0.1955 (10)	0.4656 (12)	0.0339 (18)	0.620 (13)
H11	0.0698	0.1412	0.5069	0.041*	0.620 (13)
C12	−0.0495 (10)	0.2268 (7)	0.3805 (7)	0.0453 (18)	0.620 (13)
H12A	−0.0287	0.2777	0.3348	0.054*	0.620 (13)
H12B	−0.0943	0.1702	0.3353	0.054*	0.620 (13)
C13	−0.1573 (10)	0.2667 (8)	0.4336 (8)	0.066 (2)	0.620 (13)
H13A	−0.2484	0.2890	0.3782	0.080*	0.620 (13)
H13B	−0.1844	0.2139	0.4743	0.080*	0.620 (13)
C14	−0.0905 (13)	0.3506 (7)	0.5072 (9)	0.071 (3)	0.620 (13)
H14A	−0.0680	0.4049	0.4661	0.085*	0.620 (13)
H14B	−0.1619	0.3745	0.5408	0.085*	0.620 (13)
C15	0.0478 (13)	0.3180 (11)	0.5913 (9)	0.076 (3)	0.620 (13)
H15A	0.0234	0.2671	0.6355	0.092*	0.620 (13)
H15B	0.0918	0.3739	0.6380	0.092*	0.620 (13)
C16	0.1608 (11)	0.2771 (13)	0.5433 (12)	0.0610 (18)	0.620 (13)
H16A	0.1945	0.3300	0.5063	0.073*	0.620 (13)
H16B	0.2475	0.2522	0.6009	0.073*	0.620 (13)
C11A	0.114 (2)	0.2078 (18)	0.475 (2)	0.0339 (18)	0.380 (13)
H11A	0.0753	0.1557	0.5109	0.041*	0.380 (13)
C12A	−0.0197 (17)	0.2566 (12)	0.3935 (12)	0.0453 (18)	0.380 (13)
H12C	0.0143	0.3053	0.3518	0.054*	0.380 (13)
H12D	−0.0788	0.2068	0.3438	0.054*	0.380 (13)
C13A	−0.1153 (15)	0.3069 (13)	0.4503 (13)	0.066 (2)	0.380 (13)
H13C	−0.1967	0.3420	0.3972	0.080*	0.380 (13)
H13D	−0.1595	0.2568	0.4839	0.080*	0.380 (13)
C14A	−0.030 (2)	0.3779 (12)	0.5336 (14)	0.071 (3)	0.380 (13)
H14C	0.0048	0.4325	0.4992	0.085*	0.380 (13)
H14D	−0.0945	0.4051	0.5710	0.085*	0.380 (13)
C15A	0.100 (2)	0.3296 (19)	0.6123 (14)	0.076 (3)	0.380 (13)
H15C	0.0645	0.2810	0.6532	0.092*	0.380 (13)
H15D	0.1580	0.3792	0.6628	0.092*	0.380 (13)
C16A	0.2001 (18)	0.279 (2)	0.560 (2)	0.0610 (18)	0.380 (13)
H16C	0.2474	0.3281	0.5276	0.073*	0.380 (13)
H16D	0.2787	0.2429	0.6143	0.073*	0.380 (13)
C21	0.1299 (3)	0.0773 (2)	0.2924 (2)	0.0305 (5)	
H21	0.0502	0.1198	0.2458	0.037*	
C22	0.0573 (3)	−0.0116 (2)	0.3232 (3)	0.0414 (7)	
H22A	0.1338	−0.0552	0.3695	0.050*	
H22B	−0.0080	0.0095	0.3631	0.050*	
C23	−0.0321 (4)	−0.0665 (3)	0.2234 (3)	0.0564 (9)	
H23A	−0.0759	−0.1255	0.2437	0.068*	
H23B	−0.1135	−0.0245	0.1801	0.068*	
C24	0.0637 (4)	−0.0955 (3)	0.1588 (3)	0.0574 (10)	
H24A	0.0021	−0.1279	0.0928	0.069*	
H24B	0.1382	−0.1431	0.1997	0.069*	
C25	0.1420 (4)	−0.0083 (3)	0.1301 (3)	0.0539 (9)	
H25A	0.0682	0.0356	0.0818	0.065*	
H25B	0.2088	−0.0315	0.0922	0.065*	
C26	0.2313 (3)	0.0484 (2)	0.2299 (2)	0.0384 (6)	
H26A	0.3128	0.0073	0.2746	0.046*	
H26B	0.2744	0.1076	0.2093	0.046*	

### Atomic displacement parameters (Å^2^)

**Table:**

	*U*^11^	*U*^22^	*U*^33^	*U*^12^	*U*^13^	*U*^23^
Ga1	0.02597 (13)	0.02897 (14)	0.02464 (13)	0.00414 (11)	0.00541 (10)	−0.00146 (11)
Br1	0.04072 (16)	0.03471 (15)	0.04223 (16)	−0.00386 (12)	0.00408 (13)	−0.00738 (12)
Br2	0.04296 (17)	0.0654 (2)	0.04385 (18)	0.00912 (15)	0.02185 (14)	0.01749 (16)
P1	0.0278 (3)	0.0276 (3)	0.0266 (3)	0.0028 (2)	0.0077 (2)	0.0016 (2)
C11	0.036 (3)	0.033 (4)	0.035 (3)	0.011 (3)	0.014 (2)	0.005 (2)
C12	0.040 (4)	0.046 (5)	0.048 (3)	0.018 (3)	0.011 (3)	0.003 (3)
C13	0.052 (4)	0.075 (6)	0.076 (4)	0.035 (4)	0.026 (4)	0.008 (4)
C14	0.085 (7)	0.061 (5)	0.077 (6)	0.036 (5)	0.041 (5)	−0.001 (4)
C15	0.085 (8)	0.083 (5)	0.062 (5)	0.032 (6)	0.027 (4)	−0.023 (4)
C16	0.060 (5)	0.062 (3)	0.056 (5)	0.017 (5)	0.013 (4)	−0.023 (3)
C11A	0.036 (3)	0.033 (4)	0.035 (3)	0.011 (3)	0.014 (2)	0.005 (2)
C12A	0.040 (4)	0.046 (5)	0.048 (3)	0.018 (3)	0.011 (3)	0.003 (3)
C13A	0.052 (4)	0.075 (6)	0.076 (4)	0.035 (4)	0.026 (4)	0.008 (4)
C14A	0.085 (7)	0.061 (5)	0.077 (6)	0.036 (5)	0.041 (5)	−0.001 (4)
C15A	0.085 (8)	0.083 (5)	0.062 (5)	0.032 (6)	0.027 (4)	−0.023 (4)
C16A	0.060 (5)	0.062 (3)	0.056 (5)	0.017 (5)	0.013 (4)	−0.023 (3)
C21	0.0251 (11)	0.0354 (14)	0.0257 (12)	0.0027 (10)	0.0010 (9)	0.0005 (10)
C22	0.0356 (15)	0.0427 (17)	0.0425 (16)	−0.0079 (13)	0.0084 (13)	−0.0009 (13)
C23	0.0493 (19)	0.051 (2)	0.056 (2)	−0.0158 (17)	0.0010 (17)	−0.0079 (17)
C24	0.063 (2)	0.048 (2)	0.0420 (18)	−0.0013 (17)	−0.0084 (16)	−0.0148 (16)
C25	0.057 (2)	0.071 (2)	0.0271 (15)	0.0003 (18)	0.0041 (14)	−0.0121 (16)
C26	0.0348 (14)	0.0521 (18)	0.0259 (13)	−0.0001 (13)	0.0068 (11)	−0.0040 (12)

### Geometric parameters (Å, º)

**Table:**

Ga1—Br2	2.3612 (5)	C15—H15A	0.9900
Ga1—Br1	2.3807 (5)	C15—H15B	0.9900
Ga1—P1	2.4164 (7)	C16—H16A	0.9900
Ga1—Ga1^i^	2.4353 (6)	C16—H16B	0.9900
P1—C21	1.824 (3)	C21—C22	1.526 (4)
P1—C11A	1.83 (2)	C21—C26	1.527 (4)
P1—C11	1.837 (13)	C21—H21	1.0000
P1—H1	1.31 (3)	C22—C23	1.523 (5)
C11—C16	1.516 (7)	C22—H22A	0.9900
C11—C12	1.535 (6)	C22—H22B	0.9900
C11—H11	1.0000	C23—C24	1.506 (6)
C12—C13	1.534 (7)	C23—H23A	0.9900
C12—H12A	0.9900	C23—H23B	0.9900
C12—H12B	0.9900	C24—C25	1.527 (6)
C13—C14	1.512 (10)	C24—H24A	0.9900
C13—H13A	0.9900	C24—H24B	0.9900
C13—H13B	0.9900	C25—C26	1.537 (4)
C14—C15	1.499 (9)	C25—H25A	0.9900
C14—H14A	0.9900	C25—H25B	0.9900
C14—H14B	0.9900	C26—H26A	0.9900
C15—C16	1.536 (7)	C26—H26B	0.9900
			
Br2—Ga1—Br1	107.306 (18)	C11—C16—C15	111.0 (7)
Br2—Ga1—P1	101.11 (2)	C11—C16—H16A	109.4
Br1—Ga1—P1	98.25 (2)	C15—C16—H16A	109.4
Br2—Ga1—Ga1^i^	114.05 (2)	C11—C16—H16B	109.4
Br1—Ga1—Ga1^i^	115.13 (2)	C15—C16—H16B	109.4
P1—Ga1—Ga1^i^	118.93 (2)	H16A—C16—H16B	108.0
C21—P1—C11	106.3 (4)	C22—C21—C26	111.7 (3)
C21—P1—Ga1	115.00 (9)	C22—C21—P1	111.0 (2)
C11—P1—Ga1	115.7 (4)	C26—C21—P1	110.16 (19)
C21—P1—H1	102.5 (15)	C22—C21—H21	107.9
C11—P1—H1	104.4 (16)	C26—C21—H21	107.9
Ga1—P1—H1	111.6 (15)	P1—C21—H21	107.9
C16—C11—C12	111.9 (5)	C23—C22—C21	109.6 (3)
C16—C11—P1	110.1 (8)	C23—C22—H22A	109.7
C12—C11—P1	113.1 (8)	C21—C22—H22A	109.7
C16—C11—H11	107.1	C23—C22—H22B	109.7
C12—C11—H11	107.1	C21—C22—H22B	109.7
P1—C11—H11	107.1	H22A—C22—H22B	108.2
C13—C12—C11	110.0 (6)	C24—C23—C22	110.7 (3)
C13—C12—H12A	109.7	C24—C23—H23A	109.5
C11—C12—H12A	109.7	C22—C23—H23A	109.5
C13—C12—H12B	109.7	C24—C23—H23B	109.5
C11—C12—H12B	109.7	C22—C23—H23B	109.5
H12A—C12—H12B	108.2	H23A—C23—H23B	108.1
C14—C13—C12	111.3 (6)	C23—C24—C25	112.4 (3)
C14—C13—H13A	109.4	C23—C24—H24A	109.1
C12—C13—H13A	109.4	C25—C24—H24A	109.1
C14—C13—H13B	109.4	C23—C24—H24B	109.1
C12—C13—H13B	109.4	C25—C24—H24B	109.1
H13A—C13—H13B	108.0	H24A—C24—H24B	107.9
C15—C14—C13	110.0 (6)	C24—C25—C26	111.1 (3)
C15—C14—H14A	109.7	C24—C25—H25A	109.4
C13—C14—H14A	109.7	C26—C25—H25A	109.4
C15—C14—H14B	109.7	C24—C25—H25B	109.4
C13—C14—H14B	109.7	C26—C25—H25B	109.4
H14A—C14—H14B	108.2	H25A—C25—H25B	108.0
C14—C15—C16	111.8 (6)	C21—C26—C25	109.2 (3)
C14—C15—H15A	109.2	C21—C26—H26A	109.8
C16—C15—H15A	109.2	C25—C26—H26A	109.8
C14—C15—H15B	109.2	C21—C26—H26B	109.8
C16—C15—H15B	109.2	C25—C26—H26B	109.8
H15A—C15—H15B	107.9	H26A—C26—H26B	108.3
			
Br2—Ga1—P1—C21	−99.72 (10)	P1—C11—C16—C15	−180.0 (11)
Br1—Ga1—P1—C21	150.72 (10)	C14—C15—C16—C11	−55.2 (13)
Ga1^i^—Ga1—P1—C21	25.94 (11)	C11—P1—C21—C22	−64.9 (6)
Br2—Ga1—P1—C11	24.9 (6)	Ga1—P1—C21—C22	64.5 (2)
Br1—Ga1—P1—C11	−84.6 (6)	C11A—P1—C21—C26	167.9 (11)
Ga1^i^—Ga1—P1—C11	150.6 (6)	C11—P1—C21—C26	170.9 (6)
C21—P1—C11—C16	−164.2 (8)	Ga1—P1—C21—C26	−59.8 (2)
Ga1—P1—C11—C16	66.8 (9)	C26—C21—C22—C23	−59.0 (3)
C21—P1—C11—C12	−38.2 (11)	P1—C21—C22—C23	177.6 (2)
Ga1—P1—C11—C12	−167.2 (7)	C21—C22—C23—C24	56.9 (4)
C16—C11—C12—C13	−54.2 (12)	C22—C23—C24—C25	−55.9 (4)
P1—C11—C12—C13	−179.3 (8)	C23—C24—C25—C26	55.1 (4)
C11—C12—C13—C14	56.9 (10)	C22—C21—C26—C25	57.8 (3)
C12—C13—C14—C15	−58.8 (10)	P1—C21—C26—C25	−178.3 (2)
C13—C14—C15—C16	57.5 (12)	C24—C25—C26—C21	−54.8 (4)
C12—C11—C16—C15	53.3 (13)		

Symmetry code: (i) −*x*+1, −*y*, −*z*+1.
